# Actinomycin X2, an Antimicrobial Depsipeptide from Marine-Derived *Streptomyces cyaneofuscatus* Applied as a Good Natural Dye for Silk Fabric

**DOI:** 10.3390/md20010016

**Published:** 2021-12-23

**Authors:** Wei Chen, Kaixiong Ye, Xiaoji Zhu, Huihui Zhang, Ranran Si, Jianing Chen, Zijun Chen, Kaili Song, Zhicheng Yu, Bingnan Han

**Affiliations:** 1 Department of Development Technology of Marine Resources, College of Life Sciences and Medicine, Zhejiang Sci-Tech University, Hangzhou 310018, China; 201920201070@mails.zstu.edu.cn (W.C.); kaixiongye@163.com (K.Y.); huihuizhang09@163.com (H.Z.); 202020801006@mails.zstu.edu.cn (J.C.); 202020801010@mails.zstu.edu.cn (Z.C.); 2 Engineering Research Center for Eco-Dyeing & Finishing of Textiles, Zhejiang Sci-Tech University, Hangzhou 310018, China; 201920302006@mails.zstu.edu.cn (X.Z.); kailisong@zstu.edu.cn (K.S.); yuzhicheng8@aliyun.com (Z.Y.); 3 School of Materials Science and Engineering, Zhejiang Sci-Tech University, Hangzhou 310018, China; Siranran95@163.com; 4 Key Laboratory of Advanced Textile Materials and Manufacturing Technology, Ministry of Education, Zhejiang Sci-Tech University, Hangzhou 310018, China

**Keywords:** actinomycin X2, microbial pigments, dyeing, silk, antibacterial properties

## Abstract

Actinomycins as clinical medicine have been extensively studied, while few investigations were conducted to discover the feasibility of actinomycins as antimicrobial natural dye contributing to the medical value of the functional fabrics. This study was focused on the application of actinomycin X2 (Ac.X2), a peptide pigment cultured from marine-derived *Streptomyces cyaneofuscatus*, in the dyeing and finishing of silk fabric. The dyeing potential of Ac.X2 with silk vs. cotton fabrics was assessed. As a result, the silk fabric exhibited greater uptake and color fastness with Ac.X2. Through Fourier transform infrared spectroscopy (FT-IR), scanning electron microscopy (SEM), and X-ray diffraction (XRD) analyses, some changes of chemical property for the dyed fabric and Ac.X2 were studied. The silk fabric dyed with Ac.X2 exhibited good UV protection ability. The antibacterial properties of dyed and finished silk were also evaluated, which exhibited over 90% antibacterial activity even after 20 washing cycles. In addition, the brine shrimp assay was conducted to evaluate the general toxicity of the tested fabric, and the results indicated that the dyed silk fabrics had a good biological safety property.

## 1. Introduction

Natural pigments, a surprisingly indispensable part of human life, play an important role in the food, cosmetic, medical treatment, and textile industries [1,2,3]. Since the early 20th century, a number of active natural pigments have been identified from marine sources, such as marine invertebrates, algae, and microbes. The major categories of these pigments can be divided by chemical structures, either as carotenoids, indole derivatives (quinones and violacein), alkaloids (prodiginines and tambjamines), polyenes, macrolides, peptides, or terpenoids [4,5,6,7,8,9,10]. Many of these pigments have a variety of biological activities, including antibacterial, antioxidant, and anti-inflammatory [11,12,13].

During the last few decades, a range of synthetic functional chemicals such as triclosan, metallic salts, quaternary ammonium-based products, etc. [14,15,16,17], have been developed and widely used as finishing agents for making antimicrobial silk fabric. However, these products are known to have significant limitations, such as toxic side effects, skin irritation, and effluent problems [18]. Many nature dyes with antimicrobial activity have been applied to silk textiles such as indigoids, prodigiosins, curcumins, and tannins [19,20,21]. However, most of these natural dyes applied to dyeing and finishing of silk fabrics are characterized by poor wash fastness, poor stability, and less sustainable use of bio-resources [22,23,24].

Actinomycetes, as a rich source of various secondary metabolites, have drawn more and more attention in the field of biomedicine and biotechnology [25,26,27]. Actinomycins, a class of cyclic depsipeptides, featured a phenoxazinone chromophore. Apart from their variety of biological properties, including antibacterial, antiviral, and antitumor effects [28,29,30,31], they are characterized by bright orange-yellow color pigments. During the last few years, the interest in actinomycins as clinical medicine has been gradually increased [32,33,34,35], while few investigations were conducted to discover the feasibility of actinomycins as antimicrobial natural dye contributing to the medical value of the functional fabrics.

In this study, a strain of *Streptomyces cyaneofuscatus* isolated from the cyanobacterium *Lyngbya* sp. collected from Nanji Island, China, was cultured and its main secondary metabolite actinomycin X2 was chemically and biologically characterized. This investigation was focused on the dyeing potential of Ac.X2 as a bio-colorant for silk fabric by assessing of dyeing performance of silk vs. cotton fibers. Through FT-IR, SEM, and XRD analysis, some changes of functional group with silk fabric and Ac.X2 were also studied. Finally, the antibacterial potential, UV protection ability, and the toxicity of the dyed silk fabrics were also evaluated. The outcome of this study has supported that Ac.X2 could be applied as an active natural dye for silk fabric contributing to the excellent wash fastness, strong stability as well as great antibacterial properties of the dyed silk.

## 2. Results and Discussion

### 2.1. Isolation and Identification of Pigment-Producing Actinomycetes

Actinomycetes are well known for the production of chemically and biologically diverse secondary metabolites, such as antibiotics and pigments. A strain of actinomycete producing bright orange-yellow pigment was recently isolated from marine cyanobacteria collected from Nanji Island, China. The neighbor-joining phylogenetic tree (Figure S1), based on 16S rRNA gene sequences, revealed that strain fell within the clade comprising the type strains of species of the genus Streptomyces, clustering coherently with the type strain of *Streptomyces cyaneofuscatus* with a sequence similarity of 99.6%. Based on both morphological and molecular characteristics, this strain was identified as *Streptomyces cyaneofuscatus* and deposited at the China General Microbiological Culture Collection Center (CGMCC) with the accession number.

### 2.2. Fermentation and Characterization of Actinomycin X2

**Figure 1:**
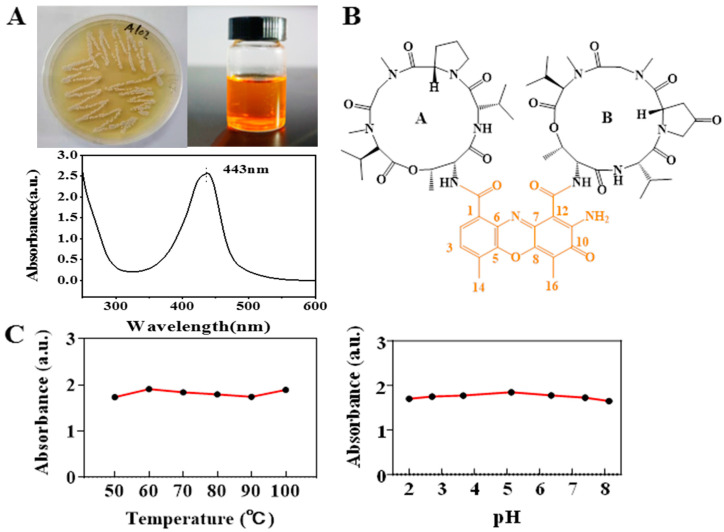
(**A**) The colony grew on G1 agar at 28 °C for 14 days; crude pigment of the liquid culture; The UV-visible spectra of actinomycin X2. (**B**) Structure of Ac.X2. (**C**) Temperature and pH stability of Ac.X2.

This strain was cultured for 7 days (pH 7.0, temperature 28 °C, rotate speed 160 rpm) in a 500 mL Erlenmeyer flask containing 200 mL ISP4 medium. A total of 200 mL fermentation broth was extracted by ethyl acetate and obtained crude orange-yellow pigment (Figure 1A). The extraction of the culture filtrates produced yields of crude pigment at 98.21 ± 2.68 mg/L with a total fermentation broth of 2 L. Through a preparative liquid chromatography, a pure orange-yellow pigment as the only major component was obtained. It was estimated that the ethyl acetate extract consists of 19.7% of pure actinomycin. This pigment was analyzed by high-resolution mass spectrometry presenting the molecular weight *m*/*z* 1269.6173 ([M + H]^+^, calcd:1269.62) (Figure S2). Combining ^1^H NMR and ^13^C NMR analysis (Figure S3), in comparison with the previously reported data [36], the structure of this pigment was confirmed to be actinomycin X2 (Ac.X2) (Figure 1B), displaying a maximum UV absorption peak at 443 nm (Figure 1A). Ac.X2 exhibited excellent thermal stability, acid and alkali resistance (Figure 1C). The antibacterial test of Ac.X2 using the paper diffusion method showed a strong inhibition effect with inhibition zones of 20 mm on *Staphylococcus aureus* [36] with a weak effect on *Escherichia coli.* (Figure S4).

### 2.3. Characterization of Dyed Textile

#### 2.3.1. Optimization of Dyeing Conditions

**Figure 2:**
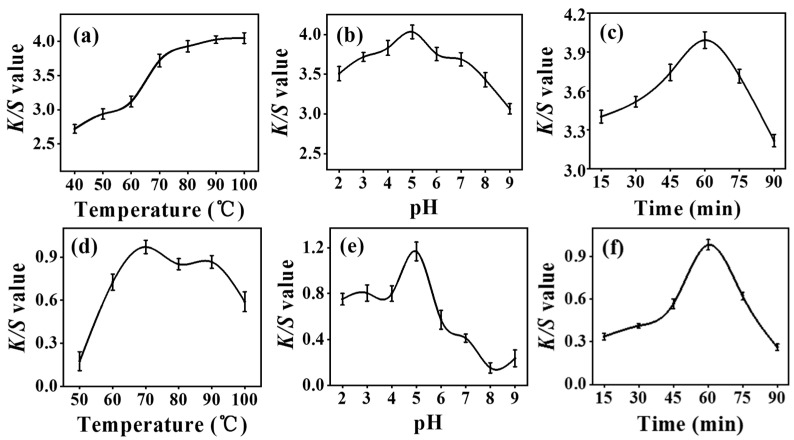
Effect of dyeing temperature on *K*/*S* value of (**a**) dyed silk; (**d**) dyed cotton. Effect of dye bath pH on *K*/*S* value of (**b**) dyed silk; (**e**) dyed cotton. Effect of dyeing time on *K*/*S* value of (**c**) dyed silk; (**f**) dyed cotton.

The exploration of optimal dyeing conditions of temperature, pH, and time was based on the method of the one-factor-at-a-time optimization technique (O.F.T.) evaluated by reflectance spectroscopy (*K*/*S*) [37]. The results of dyeing temperature optimization of silk fabric indicated that the color strength increased consistently with increasing temperature from 40 to 100 °C (Figure 2a). In contrast, the color strength of dyed cotton fabric tended to decrease after reaching the maximum at 70 °C (Figure 2d). It can be attributed to that the high temperature leading to the damage of the cellulose molecule [38]. The results of dyeing pH optimization exhibited that both silk and cotton fabric achieved the highest *K*/*S* value at pH 5 (Figure 2b,e). The results of dyeing time optimization showed that both silk and cotton fabric reached the maximum color strength when the dyeing process lasted for 60 min, and the *K*/*S* value decreased in contrast with further increase in dyeing time (Figure 2c,f). Overall, the silk fabric presented greater color strength dyed with Ac.X2 than the cotton fabric under each optimized condition.

#### 2.3.2. Color Characteristics of Dyed Fabrics

The corresponding color strengths (expressed by *K*/*S* value) and the CIELAB color coordinates were listed for silk fabric vs. cotton fabric (Table 1 and Table S1). As for the colors, it indicated that the values of the coordinates a* and b* were higher for the dyed silk fabric, suggesting that dyed silk fabrics obtained more red and yellow character [39]. The color difference (ΔE*) between the dyed fabrics exhibited that the dyed silk fabric had a better building-up capability than the cotton toward the nature dye Ac.X2. Overall, the silk fabric presented a much better uptake value of Ac.X2 with exhaustion (85.43%) compared to the value of 20.76% for the cotton fabric.

As seen in Table 2, the color shades of dyed silk showed a slight decline after 5 laundering cycles, whereas cotton fabrics had poor color retention. Moreover, it was also noted that the Δ*K*/*S* of cotton changed obviously after 5 laundering cycles but silk fabrics showed little difference, which implicated that the silk fabric might have a much stronger molecular interaction with Ac.X2 compared to the cotton fabric. Further, an N, N -Dimethylformamide (DMF) stripping study was performed to determine the interacting strength of Ac.X2 with silk vs. cotton fabrics. The dyed silk fabric withstood the treatment of DMF much stronger than the dyed cotton, which displayed by the Δ*K*/*S* values in Table 2. This result suggested that intermolecular covalent bonding may occur during the dyeing process of Ac.X2 with silk fabric [24,40].

#### 2.3.3. Fourier Transform Infrared Spectroscopy (FT-IR) Analysis

**Figure 3:**
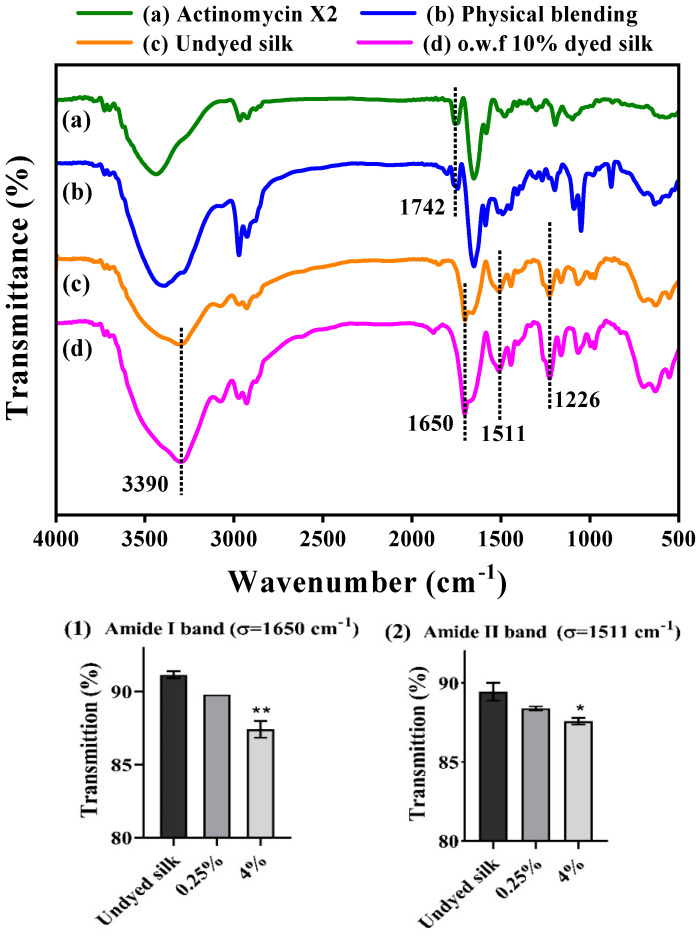
FT-IR images of (a) actinomycin X2, (Ac.X2), (b) physical blending, (c) undyed silk, (d) o.w.f 10%-dyed silk fabric. Transmittance of (1) amide I band, (2) amide II band. * *p* < 0.05, ** *p* < 0.01.

The structural functionalization of the dyed silk in comparison to the undyed silk and Ac.X2 was analyzed by the FT-IR technique (Figure 3). The FT-IR analysis of the Ac.X2 and physical blending material revealed the appearance of an absorption band at 1742 cm^−1^, characteristic of the C=O ester stretch band [41], but this band was not observed in the spectra of the dyed silk fabrics, which implicated that the ester functionalization of Ac.X2 did not exist in the dyed silk fabrics. The absorption spectra of the undyed silk and the dyed silk with o.w.f 10% Ac.X2 exhibited a similar vibration pattern, in which a major peak at 3279 cm^−1^, assigned to the -N-H stretching vibration, C=O (amide I band indicative of β-sheet) at 1650 cm^−1^ and N-H deformation (amide II band indicative of β-sheet) at 1511 cm^−1^ were observed in both dyed and undyed silk fabrics [42,43,44]. However, the absorption intensity of the above characteristic signals of peptide functionalities increased in the dyed sample compared to the undyed silk fabric (Figure 3(1,2)).

#### 2.3.4. X-ray Diffraction (XRD) Analysis and Scanning Electron Microscopy (SEM) Analysis

**Figure 4:**
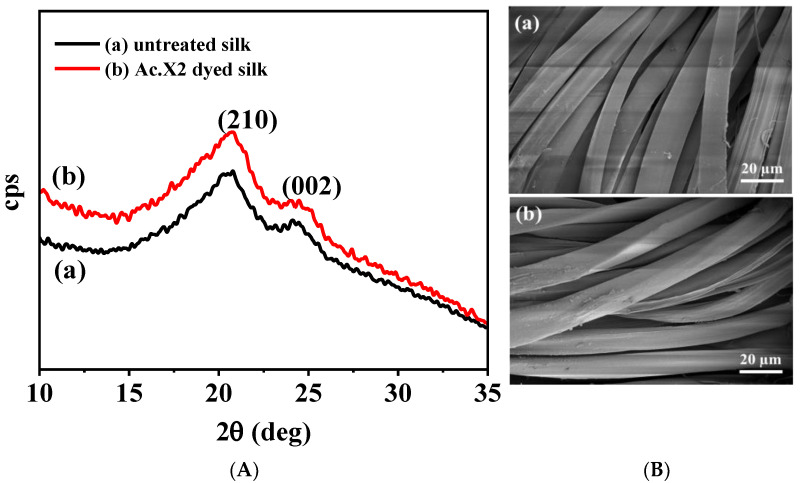
(**A**) XRD patterns of undyed and o.w.f 10% Ac.X2-dyed silk fabrics. (**B**) SEM images (1000X), (**a**) undyed silk fabric, (**b**) Ac.X2-dyed silk fabric.

XRD analysis of Ac.X2-dyed silk and untreated silk was carried out for the purpose of determining the change in the secondary structure of silk [45], as shown in Figure 4A. The similar diffraction patterns of the 2θ angles were observed in the dyed and undyed silk fabrics, in which two obvious crystalline peaks characteristics of the protein β-sheet structure, were labeled by the (210) and (002) planes at 2θ of 19.4° and 24.2°, appeared, respectively [46,47]. As the results, it also suggested that the dyeing process of Ac.X2 mainly occurred in the amorphous area of the silk fiber rather than the crystalline area.

The effect of the coloration process on the silk fabric morphology was examined by SEM micrograph. As shown in Figure 4B, both dyed and undyed silk fabrics were observed to be smooth, uniform, and free from roughness with no particle deposition and surface irregularity in morphology. Therefore, it might implicate the penetration of the Ac.X2 into the fiber structure rather than a surface deposition, resulting in strong fastness properties of the dyed silk fabric.

### 2.4. Fastness and Functional Finishing Properties of the Silk Fabrics Dyed with Ac.X2

#### 2.4.1. Color Strength Associated with Dye Concentration

**Figure 5:**
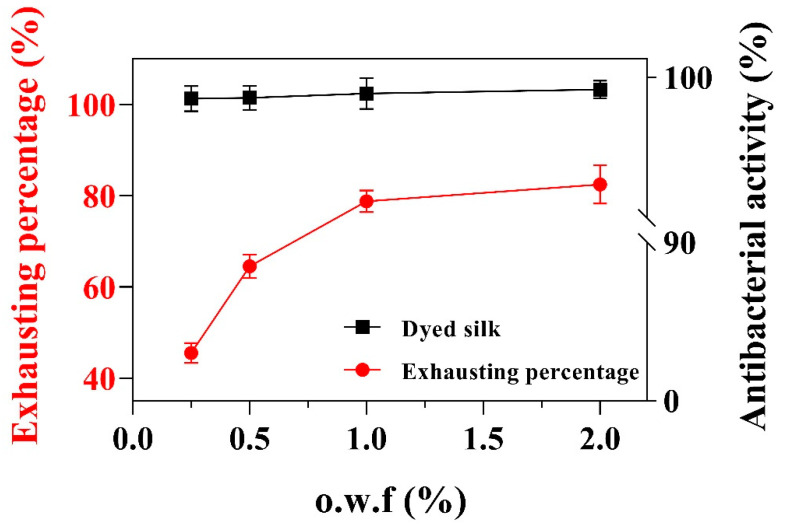
UPF (bars) and UVA transmittance (black dots) for undyed and different dye concentrations of Ac.X2-dyed silk fabrics. Bars are means of three measurements. Horizontal black line represents the UVA transmittance threshold established by the European standard for sun protective clothing (** *p* < 0.01).

To further explore the dyeing performance of Ac.X2 on the silk fabric associated with their antibacterial activities, six different dye concentrations of Ac.X2 (o.w.f 0.1%–4%) were evaluated. As seen in Table 3, the color strength of dyed silk fabrics was increased with dye concentration in a reasonably good manner. From the colorimetric data (a*/b* plots) depicted in Table 3, it can be seen that the yellow shade from Ac.X2 produced varying color saturation with increasing a* and b* values due to the improving adsorption of dyed silk fabric, and their shades present reddish-yellow had a little shift toward red-coordinate in the yellow-red zone of color space. The adsorption quantity increased with increasing initial concentration, but the color depth of dyed silk had little augmentation when its initial concentration exceeded 1% o.w.f (Figure 5). These results are consistent with those reported by Zhou et al. [48], who stated that the uptake of *Rheum emodi* by silk displayed the extent of exhaustion over 40%, but no increase in its adsorption when the concentration exceeded 4% o.w.f.

#### 2.4.2. Color Fastness

Silk fabrics are subjected to frequent washing, rubbing, and perspiring during their usage. Table 4 shows the color fastness (wash, rub, and perspiration fastness) results of the o.w.f 1% Ac.X2-dyed silk fabrics under the optimum dyeing process. The fastness ratings on a gray scale ranging from 1 to 5 were evaluated (1 for bad, 2 for fairly good, 3 for good, 4 for very good, and 5 for excellent results). The grade of color change to wash was “very good” (4), and the color staining to the wash of the dyed silk fabrics was rated as “excellent” (5). As shown in Table 4, the perspiration and rub fastness of silk fabric dyed in the optimum condition were considered good to excellent range, quite satisfactory for industrial-scale dyeing, with a general rating of 4–5 on a gray scale. Owing to the absence of direct affinity between most natural dyes and fibers, many environment-unfriendly mordants (e.g., alum, copper sulfate, and ferrous sulfate) are needed to help to obtain good fastness for natural dyes with fabrics [42,49,50]. However, Ac.X2 exhibited excellent fastness to silk without using mordant, which may indicate good potential for industrial application.

#### 2.4.3. UV Protection Performance

**Figure 6:**
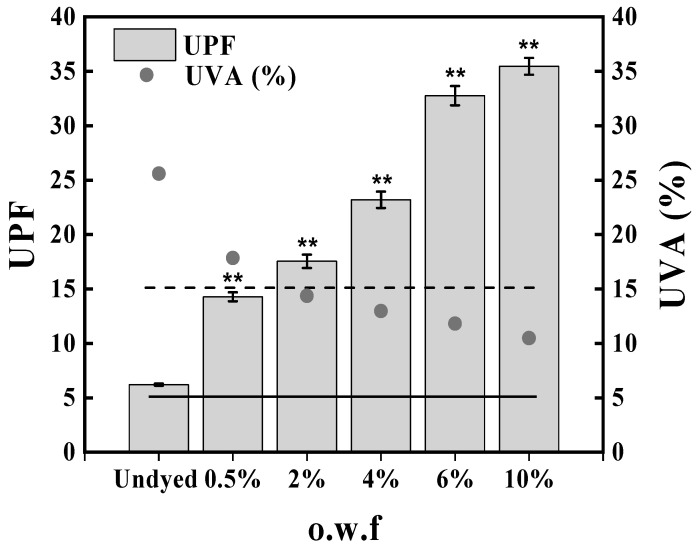
Antibacterial activity and exhausting percentage of different o.w.f-dyed silk against *Staphylococcus aureus* tested by GB/T 20944.3-2008 method.

In order to avoid or limit the health risks such as sunburn, premature aging, allergies, and skin cancers, it is important to reduce UV ray exposure with clothing made of protective materials. UV protection levels of silk fabric samples dyed with different dye concentrations are presented in Figure 6. As regards the UPF, the dyed silk fabric over 0.5% o.w.f reaches values corresponding to the good protection category in the undyed fabrics. However, silk fabrics dyed with Ac.X2 showed a poor protection level for UVA transmittance with values below 5% (Figure 6). This is the threshold above which photosensitive skin disorders, such as chronic actinic dermatitis and solar urticaria, can be aggravated according to the European standard for sun protective clothing and also the Chinese National standard GB/T18830-2009 [51,52]. Silk fabrics dyed with baicalin extract dye and curcumin also exhibited good UPF values, which can be classified as a ‘very good’ scale [20]. Similar results were found for marigold extract dye, honeysuckle extracts, and Chinese tallow leaves extract on wool fabrics, and chitosan mordanted green tea dye on cotton fabrics [53,54,55]. In this perspective, it can be concluded that dyed fabrics are more protective than undyed ones and the protection level rises with the increase in dye concentration.

#### 2.4.4. Antibacterial Activity and Toxicity of Brine Shrimp

Silk fabrics are especially susceptible to microbial growth with poor antibacterial activity, and Ac.X2 has been investigated to have good antibacterial potential. Thus, two methods of AATCC 100-2012 (Figure S7) and GB/T 20944.3-2008 (Figure 5) were used to evaluate the antibacterial properties accomplished by the dyed silk fabrics. The results indicated that both methods exerted high antibacterial activity over 95% against *S. aureus* of the 0.25–2% o.w.f processed fabrics. The adsorption of Ac.X2 on silk fabrics is an alternative way to introduce the antibacterial functionality of marine pigment for their use in protective clothing and medical textiles.

**Figure 7:**
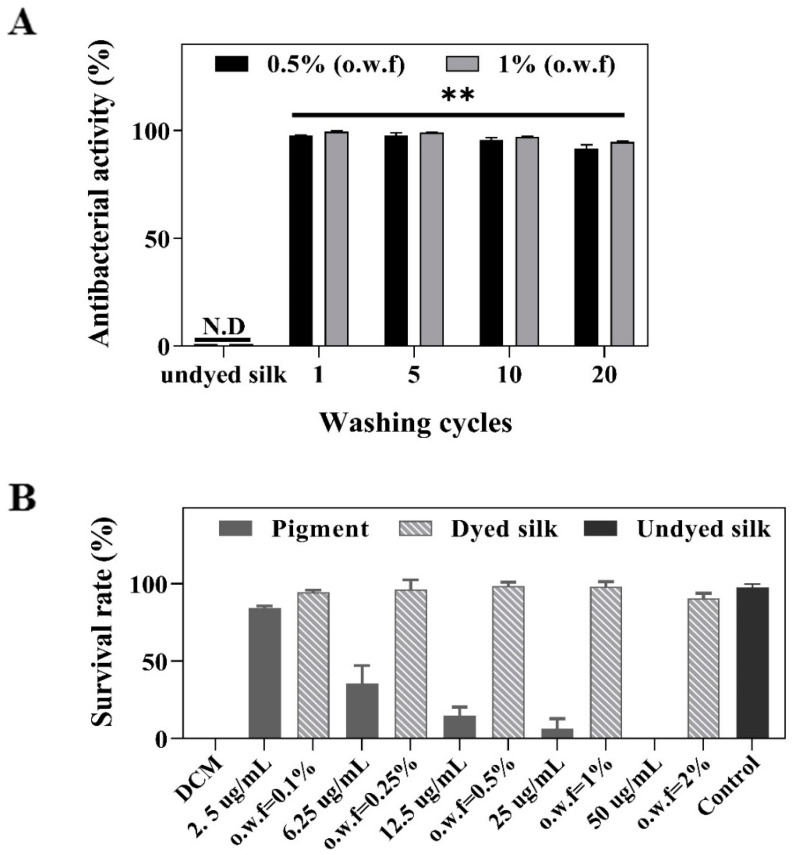
(**A**) Antibacterial activity of o.w.f 0.5%- and o.w.f 1%-dyed silk samples against *Staphylococcus aureus* tested by GB/T 20944.3-2008 method after repeated laundering. (** *p* < 0.01), N.D = No detect (**B**) The survival rate of *A. salina* with different pigment concentrations and the same amount of Ac.X2-dyed silk.

The bacteriostasis rate of the 0.5% (o.w.f)- and 1% (o.w.f)-dyed silk after long washing cycles test was presented in Figure 7A, and all samples exhibited over 90% antibacterial activity even after 20 washing cycles. The results suggested that the Ac.X2-dyed fabrics had durable antibacterial properties compared to the undyed silk, which indicates the application of Ac.X2 in silk fabric solves the limitations in terms of efficacy and durability of natural antimicrobial agents [6]. The investigation of brine shrimp toxicity was also conducted to explore the primary safety of dyed silk fabrics. The lethality assay of brine shrimp *A. salina* was developed by Vanhaecke et al. [56], which was suggested to evaluate the toxicity of compounds [57,58,59]. It showed that the pigment concentration negatively correlated with the survival rate of *A. salina*. In contrast, *A. salina* still had a high survival rate in the same amount of pigment dyed with silk fabrics groups (Figure 7B). It may implicate that the safety of the dyed fabric was almost equivalent to the normal fabric.

The public’s mature demand in recent times boomed the silk textile industry for the use of natural colorants, while the synthetic colorants with high dye exhaustion over 80% [60] have even been withdrawn from external usage due to its apparent hazards. On the other hand, the pigments such as *Terminalia catappa*, *Vaccinium Corymbosum* L. derived from raw plant materials are known to have low absorption (10%) vs. Ac.X2 (85.43%) in the silk fabric dyeing process and poor color fastness range 1–2 compared to microbial pigment Ac.X2 with the color fastness range of 4–5 [61,62]. More importantly, some natural pigments such as *Carthamus tinctorius* L. had little antibacterial activity of 29% against *S. aureus* in the absence of synthetic functional chemicals, even though the AgNPs-treated fabrics dyed with caffeic acid was highly durable as more than 90% inhibition after 10 washing cycles [63], Ac.X2 has good antibacterial activity over 90% against *S. aureus* at the low concentration of 0.5% without any additional finishing agents.

## 3. Materials and Methods

### 3.1. Textile Material

The crepe de Chine silk fabric (warp and weft count, 23 dtex/2; warp density 54 threads/cm, weft density 41 threads/cm; 70 g/m^2^ weight), the scoured and bleached cotton (warp 180 dtex, weft 180 dtex; warp density 93 yarns per inch, weft density 41 yarns per inch; 240 g/m^2^ weight), were obtained from a local ordinary mill (High Fashion Co., Ltd., Hangzhou, China).

### 3.2. Strain Isolation

Actinomycetes producing yellow pigment were isolated from cyanobacteria (Lyngbya species) collected from the shore of Nanji Island, China. Weighed 1 g of fresh cyanobacteria and cut it into small pieces (5 mm length). The cut samples were mixed with 10 mL ddH_2_O and diluted 10-fold to the concentration of 10^−1^, 10^−2^, 10^−3^, respectively. Using Gauze’s No. 1 (G1) agar medium as selective medium consisting of soluble starch 2.0%, KNO_3_ 0.1%, K_2_HPO_4_ 0.05%, MgSO_4_·7H_2_O 0.05%, NaCl 0.05%, FeSO_4_·7H_2_O 0.001%, sea salt 2.0%. Plates were incubated at 28 °C for 7 days [64].

### 3.3. Identification of Pigment-Producing Actinomycetes

#### 3.3.1. Morphological Studies

The pigment-producing strain was cultured on G1 at 28 °C for 14 days. Colony characteristics, including shape, size, and pigment-producing time were recorded. The morphological characteristics of spores and mycelia were observed by a light microscope (Nikon ECLIPSE E100, TKY, Japan).

#### 3.3.2. Molecular Studies

Genomic DNA of pigment-producing bacteria was extracted by a bacterial DNA kit (Beijing Biomed Gene Technology Co., Ltd., Beijing, China). Universal primers 27F and 1492R were used to amplify the 16S rDNA. Polymerase chain reaction (PCR) was performed in 25 μL reaction containing 0.5 μL DNA template, 0.5 μL of each forward and reverse primers, 12.5 μL 2× Mix (CoWin Biosciences, Jiangsu, China) and 11 μL deionized water, and the following conditions: initial denaturation at 94 °C for 5 min, 30 cycles of denaturation (94 °C, 1 min), annealing (55 °C, 1 min), and elongation (72 °C, 1 min 15 s), and final elongation at 72 °C for 10 min. BLAST of the NCBI database was used to carry out multiple sequence alignment. The phylogenetic tree of pigment-producing bacteria was constructed by the neighbor-joining method of MEGA 5.0.

### 3.4. Extraction and Purification of Pigment

Single colonies growing in G1 agar medium were transferred to a 250 mL Erlenmeyer flask containing 50 mL of seed medium (with the same ingredients as G1) and incubated at 28 °C and 160 rpm for 8 days. Transfer 1 mL of seed solution to a 500 mL Erlenmeyer flask containing 200 mL ISP4 medium (10 g/L soluble starch, 1 g/L MgSO_4_·7H_2_O, 2 g/L peptone, 1 mg/L FeSO_4_·7H_2_O, 1 g/L K_2_HPO_4_, 1 g/L NaCl, 2 g/L CaCO_3_, 1 mg/L MnCl_2_·7H_2_O, 1 mg/L ZnSO_4_·7H_2_O, 20 g/L sea salt, pH = 7.2–7.4) and fermented for 7 days at 28 °C and 160 rpm. After the fermentation, the fermentation broth was centrifuged (8000 rpm at 4 °C) for 10 min by an ultrafast centrifuge (BIORIDGE TG16, Shanghai, China) to collect the supernatant. Extract with ethyl acetate:fermentation broth = 1:1 volume ratio. Repeat this step three times. The extraction solution was reduced to concentrate at room temperatures, and the pigment extract was obtained. The crude pigment extract was dissolved in methanol and filtered through a membrane of 0.22 µm. The pigment was purified by HPLC with a column of YMC-Pack ODS-A (250 × 20 nm), using 75% acetonitrile as a mobile phase.

### 3.5. Characterization of Pigment

#### 3.5.1. Temperature Stability

Take 6 tubes of 5 mL 1 mg/mL solution of pigment methanol, respectively. Place the test tube in a water bath pot and heat it at 50, 60, 70, 80, 90, and 100 °C for 10 min and then cool it to room temperature. Under the maximum absorption wavelength of 443 nm, the absorbance of methanol solution was measured by spectrophotometer (MAPADA UV-1800PC) with methanol as the reference solution, and the color change was observed.

#### 3.5.2. pH Stability

The pH values were adjusted to 2, 3, 4, 5, 6, 7, and 8 by 1 M HCl and 1 M NaOH, respectively. Under the maximum absorption wavelength of 443 nm, the absorbance of methanol solution was measured by ultraviolet spectrophotometer with methanol as the reference solution, and the color change was observed.

#### 3.5.3. Antimicrobial Activity

The antimicrobial activity of pigment against *Staphylococcus aureus* (*S. aureus*, ATCC 6538, Gram-positive) and *Escherichia coli* (*E. coli*, ATCC 8739, Gram-negative) bacteria was determined by paper disk diffusion assay. The pigment was prepared into a solution of 0.1 mg/mL with dimethyl sulfoxide (DMSO). Five microliters of pigment were added to the filter paper of Luria-Bertani medium (LB), and two microliters of DMSO were used as the negative control. The LB medium was cultured in a 37 °C incubator for 24 h, and the diameter of the inhibition zone was measured.

### 3.6. Textile Dyeing

The dyeing effect of pigment on silk and cotton fabrics was evaluated. Prepare 4 × 10 cm of two kinds of each fabric and record the weight, according to 1% o.w.f (on the weight of fiber) to prepare the corresponding pigment. According to the pigment/dispersing agent NNO (2-Naphthalenesulfonic acid) (Aladdin, Shanghai, China) = 2/1 mixed to fully grind, material to liquor ratio (MLR) 1:30. CH_3_COOH and NaOH were used for adjustment of the dyebath pH to the initial values. The dyeing process was carried out in a dyeing apparatus equipped with two 500 mL steel beakers. The optimal dyeing conditions of Ac.X2 natural dye onto silk and cotton fabric were investigated with simple water bath dyeing conditions under continuous manual stirring by optimizing dyeing variables including pH (2–9), temperature (40–100 °C), and time (15–90 min) using one-factor-at-a-time (OFT) optimization technique by reflectance spectroscopy (*K*/*S*). After dyeing, rinse the samples with distilled water at 40 °C for 20 times and dry them. The fabric with the best dyeing effect was selected for further experiments.

### 3.7. Measurement

#### 3.7.1. Fourier Transform Infrared Spectroscopy (FT-IR) Analysis

The FT-IR spectra of the untreated and Ac.X2-dyed silk fabrics were recorded using an FT-IR spectrometer (Nicolet iS20, MA, USA) with a resolution of 4 cm^−1^ by recording 32 scans in % transmittance mode in the range of 500–4000 cm^−1^. The samples were prepared by using a KBr-disk.

#### 3.7.2. X-ray Diffraction (XRD) Analysis

XRD measurement of the untreated and Ac.X2-dyed silk samples was carried out on a Bruker D8 ADVANCE in the 2θ angle ranging from 10° to 35° with 0.09° step interval. The generator voltage was kept at 40 kV, and the generator current was 40 mA. The sample in the form of chopped fibers was prepared and placed on the stub.

#### 3.7.3. Scanning Electron Microscopy (SEM)

Morphological investigations on silk fabric samples colored with the actinomycin X2 for comparison with a reference silk fabric were carried out by a SU 8010 SEM, at an acceleration voltage of 30 kV, 50 pA of the current probe, and 30 mm working distance. The samples were mounted on aluminum specimen stubs with conductive plastic. Samples were sputter-coated with a 20–30 nm thick gold layer in rarefied argon, using a sputter coater, using a current intensity of 15 mA, for 2 min.

### 3.8. Characterization of the Dyed Samples

#### 3.8.1. The Influence of Dye Concentration on the Dyeing Effect

In previous experiments, the pigment showed the best dyeing effect on the silk fabric. In this experiment, silk fabric was dyed with 5 different o.w.f (0.1%, 0.2%, 0.5%, 1%, 2%, and 4%) under optimum dyeing condition.

#### 3.8.2. Color Characteristics

The color strength (*K*/*S* value) and CIE L*, a*, b* of dyed fabrics were measured by a Datacolor 600 spectrophotometer under illuminant D65, by a 10° standard observer. Each sample was folded twice so as to provide a thickness of four layers. The reflectance (R) values were converted to corresponding color strength values using Kubelka–Munk Equation (1). The CIE system was a specialized color system designed by International Commission on illumination. L* presents a lightness value (a higher lightness value represents a lower color yield); a* presents redness-greenness (+value = red, −value = green); b* presents yellowness-blueness (+value = yellow, −value = blue) [50,65,66]. The exhausting percentage of dyed silk and cotton was obtained according to Equation (2), which was used to characterize the dye uptake after the dyeing process.

*K*/*S* = (1 − R^2^) / 2R (1)

where *K*, R, and *S* are the adsorption coefficient, reflectance of dyed fabric, and the scattering coefficient, respectively.

Exhausting percentage (%) = (A_1_ − A_0_)/A_1_ × 100% (2)

where A_1_ and A_0_ are the absorbances of dye liquor at 443 nm before and after the dyeing process.

#### 3.8.3. Color Fastness Testing

The washing color fastness of dyed silk fabrics was evaluated according to the standard GB/T3921-2008 by washing the samples for 30 min at 40 °C with 0.2% (*w*/*v*) standard detergent. Dry and wet rub fastness was tested using the standard of GB/T3920-2008. The perspiration fastness was tested on the basis of GB/T3922-2013.

#### 3.8.4. UV Protection Performance

The UV protection factor (UPF) of undyed silk and different dye concentration dyed silk was determined on three fabric samples (2 × 2 cm^2^). Each sample was taken from the center of the fabric, fixed on a common slide frame, and placed in a UV-2000F Textiles UV Factor Tester (Labsphere company, NES, USA) equipped with an integrating sphere to measure both direct and diffuse transmitted light. Each sample was positioned at right angles to the light beams. Transmission measurements were made in the 290–400 nm range with a 1 nm step.

#### 3.8.5. Antibacterial Activity

The antibacterial experiment of dyed silk and after washing samples was carried out according to the American Association of Textile Chemists and Colorists AATCC 100-2012 and the antibacterial standard for textiles (GB/T 20944.3-2008). *Staphylococcus aureus* (ATCC6538) was selected as a test strain.

In the AATCC 100-2012 experiment, the test samples were inoculated with a suitable concentration of bacteria, transferred to a constant temperature incubator at 37 °C for 12 h, and then neutralized with 0.03 mol/L PBS buffer solution for 1 min. Dilute to a suitable concentration by 10 times dilution. Transfer 1 mL of the diluted solution to a clean plate, and about 15 mL of NB agar medium was irrigated. After the agar was cooled and solidified, the plates were placed in a constant temperature incubator at 37 °C for 48 h. The plates with appropriate colony density were counted, and the inhibition rate was calculated according to Equation (3):

R (%) = (A − B)/A × 100%(3)

where R is the bacterial inhibition rate (%) and A and B are the number of bacterial colonies from undyed silk and dyed silk, respectively.

In the GB/T 20944.3-2008 experiment, small pieces of fabric sample (2 × 2 cm) were introduced into 5 mL 0.03 mol/L PBS, which contained 1 × 10^5^ CFU/mL bacteria, followed by shaking incubation at 37 °C for 24 h. The medium was diluted 10 times with 0.03 mol/L PBS to a suitable multiple. Transfer 1 mL of the diluted solution to a clean plate, and about 15 mL of NB agar medium was irrigated. After the agar was cooled and solidified, the plates were placed in a constant temperature incubator at 37 °C for 48 h. The plates with appropriate colony density were counted, and the inhibition rate was calculated according to Equation (3), too. Data were treated by one-way analysis of variance (ANOVA) and multiple comparisons analyses. Statistically significant values were the ones having *p* < 0.05.

#### 3.8.6. Brine Shrimp Toxicity Assay

The lethality assay of brine shrimp Artemia salina (*A. salina*) was developed by previous studies, which had been suggested to evaluate the toxicity of compounds. Commercially available *A. salina* or brine shrimp cysts were purchased and cultivated in 3.2% of saline water. Before cultivation, the saline was aerated, and then cysts were kept at room temperature for 24 h. For toxicity screening, hatched larvae were collected and introduced in saline water. Add 0.9% saline water and approximately the same number of larvae 30 per well to make a 12-wells test culture plate. Ac.X2 with 2.5 µg/mL, 6.25 µg/mL, 12.5 µg/mL, 25 µg/mL and 1 × 1 cm dyed silk fabrics (o.w.f = 0.1%, 0.25%, 0.5%, 1%, 2%) were added to the test culture plate, while the equal volume of dimethyl sulfoxide (DMSO) (Aladdin, Shanghai, China) and dichloromethane (Aladdin, Shanghai, China) were added as blank control test and positive control separately. After 24 h at 25 °C, the percent of survival of *A. salina* was calculated.4. Conclusions

This study has demonstrated for the first time the application of actinomycin X2 (Ac.X2), a peptide orange-yellow pigment as a natural dye from marine microorganisms. By using OFT, a series of dyeing experiments was performed, and dyeing parameters were optimized, and it displayed fantastic dyeing performance with silk fabric owing to the high dye uptake percentage (85.43%) and satisfactory color fastness. The dyed silk fabric characterized by FT-IR, XRD, and SEM analysis may indicate some chemical reaction occurring under the dyeing condition. In addition, it was illustrated in this research that Ac.X2-dyed silk fabrics exerted good UV protection ability and high antibacterial activity over 95–99% against *S. aureus*, and it still had remarkable antibacterial activity (over 90%) after 20 times repeated laundering compared to other natural dye. Furthermore, brine shrimp toxicity results indicated that Ac.X2-dyed silk exhibited similar biological safety as regular silk. With the enhanced awareness of eco-safety, Ac.X2 as the marine source of natural pigment is an interesting route and offers opportunities for potential bio-based value.

## Figures and Tables

**Table 1:** Color strength, colorimetric parameters, and color difference (ΔE*) of o.w.f 1%-dyed fabric samples under previous dyeing process.

Fabric Sample	Apparent Color	*K*/*S* Value	L*	a*	b*	ΔE*	Percentage Exhaustion (%)
Silk	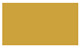	4.0740	−15.57	14.41	55.36	64.10	85.43%
Cotton	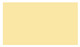	1.1094	−10.58	10.46	38.51	50.44	20.76%

*K*/*S*: Color strength, L*: lightness, a*: (+value = red, −value = green) b*: (+value = yellow, −value = blue).

**Table 2:** Ac.X2-dyed silk and cotton experienced DMF extraction and after 5 times laundering, respectively.

Fabric	After 5 Times Laundering		Δ*K*/*S*	After DMF Extraction		Δ*K*/*S*
	A.C.	*K*/*S* Value		A.C.	*K*/*S* Value	
Silk	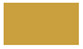	3.7444	−0.3296	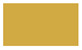	3.7529	−0.3211
Cotton	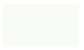	0.0464	−1.063	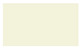	0.0689	−1.0405

A.C. = Apparent color.

**Table 3:** The effect of dye concentration on color characteristics of dyed silk fabric.

o.w.f.	Apparent Color	*K*/*S* Value	L *	a *	b *
0.1	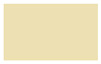	0.3657	−5.17	0.66	24.85
0.25	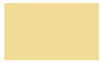	0.6665	−6.07	0.82	28.86
0.5	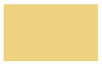	1.1644	−8.51	2.26	35.37
1	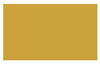	4.0740	−15.57	14.41	55.36
2	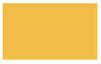	5.3555	−16.37	18.37	57.4
4	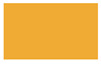	5.8977	−20.71	18.7	57.62

**Table 4:** Fastness properties of silk fabrics dyed with Ac.X2.

Wash Fastness			Rub Fastness		Perspiration Fastness					
					Acid			Alkali		
c.c.	s.s.	s.c.	Dry	Wet	c.c.	s.s.	s.c.	c.c.	s.s.	s.c.
4	5	5	4–5	4	4–5	3–4	4	4–5	3–4	4

c.c. = color change s.s. = color staining of silk s.c. = color staining of cotton.

## Supplementary Materials

The following are available online at https://www.mdpi.com/article/10.3390/md20010016/s1, Figure S1: Neighbor-joining phylogenetic tree of strain A102 based on the 16S rRNA gene sequence generated by Mega5.0, Numbers on branch nodes are bootstrap values (expressed as percentage of 1000 replications). Figure S2: HPLC and MS data of pigment. Figure S3: 13C-NMR and 1H-NMR (at 400 MHz in CDCl3) profiles of the pigment. Figure S4: Antimicrobial activity of the pigment to two microorganisms. Figure S5: Pigment absorbance standard curve. Figure S6: Effects of liquid medium on (A) different liquid medium; (B) nitrogen source; (C) carbon source; (D) pH. Figure S7: Bacteriostatic rates of different o.w.f silk tested by AATCC 100-2012 method. Figure S8: The images of plates of different o.w.f dyed silk against Staphylococcus aureus tested by AATCC 100-2012 method. Figure S9: The images of plates of different o.w.f dyed silk against Staphylococcus aureus tested by GB/T 20944.3-2008 method. Figure S10: Antibacterial activity of (A) dyed o.w.f 0.5% and (B) o.w.f 1% silk sample against Staphylococcus aureus tested by GB/T 20944.3-2008 method after different washing times. Table S1: Antibacterial activity of dyed o.w.f 0.5% and o.w.f 1% silk sample against Staphylococcus aureus tested by GB/T 20944.3-2008 method after different washing cycles.
