# Supplementary material for: Actinomycin X2, an Antimicrobial Depsipeptide from Marine-Derived Streptomyces cyaneofuscatus Applied as a Good Natural Dye for Silk Fabric
Source: Mar Drugs. 2021 Dec 23;20(1):16. doi: 10.3390/md20010016 (PMC8778624; doi:10.3390/md20010016)
Supplement: Supplementary file 1 [file marinedrugs-20-00016-s001.zip › marinedrugs-1520967-supplementary.pdf]

Supporting Information

Actinomycin X2, an antimicrobial depsipeptide from marine-derived *Streptomyces cyaneofuscatus* applied as a good natural dye for silk fabric

Wei Chen [a], Kaixiong Ye [a], Xiaoji Zhu [c], Huihui Zhang [a], Ranran Si [b], Jianing Chen [a], Zijun Chen [a], Kaili Song [c,d], Zhicheng Yu [c,d], Bingnan Han [a,*]

a Department of Development Technology of Marine Resources, College of Life Sciences and Medicine, Zhejiang Sci-Tech University, Hangzhou 310018, China

b School of Materials Science and Engineering, Zhejiang Sci-Tech University, Hangzhou 310018, China

c Engineering Research Center for Eco-Dyeing & Finishing of Textiles, Zhejiang Sci-Tech University, Hangzhou, Zhejiang 310018, China

d Key Laboratory of Advanced Textile Materials and Manufacturing Technology, Ministry of Education, Zhejiang Sci-Tech University, Hangzhou, Zhejiang 310018, China

E-mail: * Corresponding author: hanbingnan@zstu.edu.cn (B.Han)

Phone: 18057133438 (B.Han)

Contents

1. **Figure** 3

Figure S1 Neighbor-joining phylogenetic tree of strain A102 based on the 16S rRNA gene sequence generated by Mega5.0, Numbers on branch nodes are bootstrap values (expressed as percentage of 1000 replications). 3

Figure S2 HPLC and MS data of pigment 4

Figure S3 ^{13}C-NMR and ^{1}H-NMR (at 400 MHz in $CDCl_3$) profiles of the pigment. 5

Figure S4 Antimicrobial activity of the pigment to two microorganisms 5

Figure S5 Pigment absorbance standard curve. 6

Figure S6 Effects of liquid medium on **(A)** different liquid medium; **(B)** nitrogen source; **(C)** carbon source; **(D)** pH. 7

Figure S7 Bacteriostatic rates of different o.w.f silk tested by AATCC 100-2012 method. 8

Figure S8 The images of plates of different o.w.f dyed silk against *Staphylococcus aureus* tested by AATCC 100-2012 method 9

Figure S9 The images of plates of different o.w.f dyed silk against *Staphylococcus aureus* tested by GB/T 20944.3-2008 method 10

Figure S10 Antibacterial activity of (**A**) dyed o.w.f 0.5% and (**B**) o.w.f 1% silk sample against *Staphylococcus aureus* tested by GB/T 20944.3-2008 method after different washing times. 11

2. **Table** 12

Table S1 Antibacterial activity of dyed o.w.f 0.5% and o.w.f 1% silk sample against *Staphylococcus aureus* tested by GB/T 20944.3-2008 method after different washing cycles. 12

1. Figure

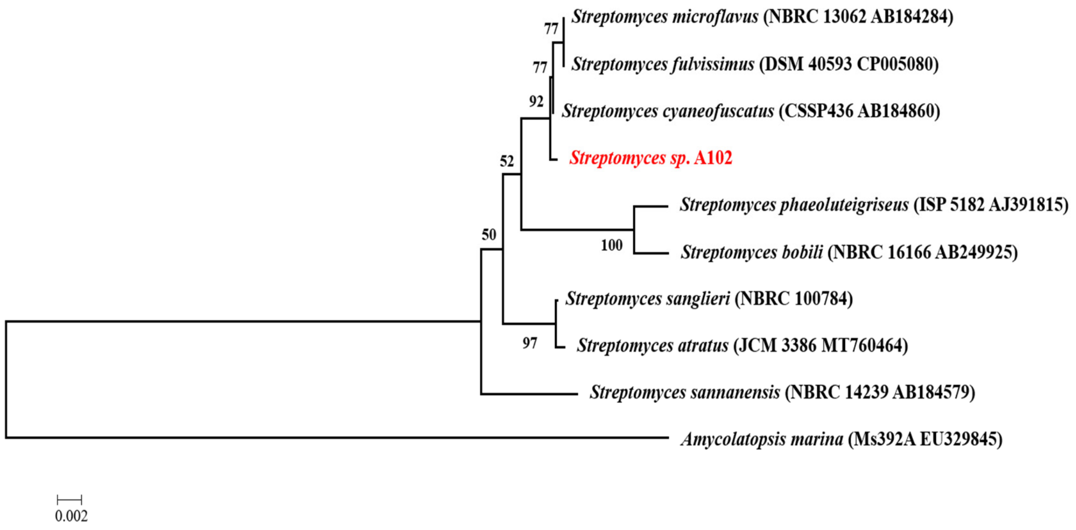

Figure S1. Neighbor-joining phylogenetic tree of strain A102 based on the 16S rRNA gene sequence generated by Mega5.0, Numbers on branch nodes are bootstrap values (expressed as percentage of 1000 replications).

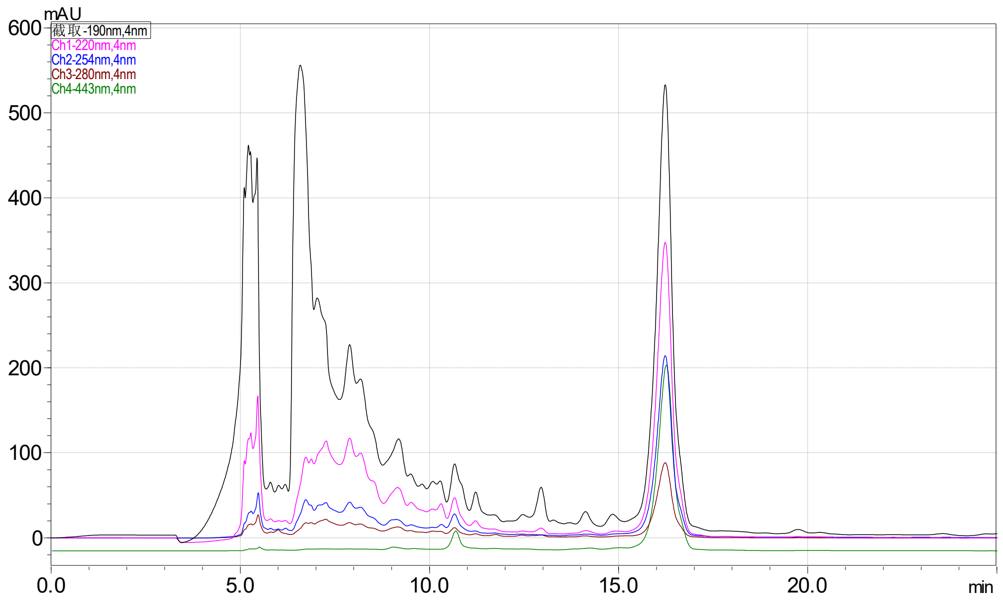

a1

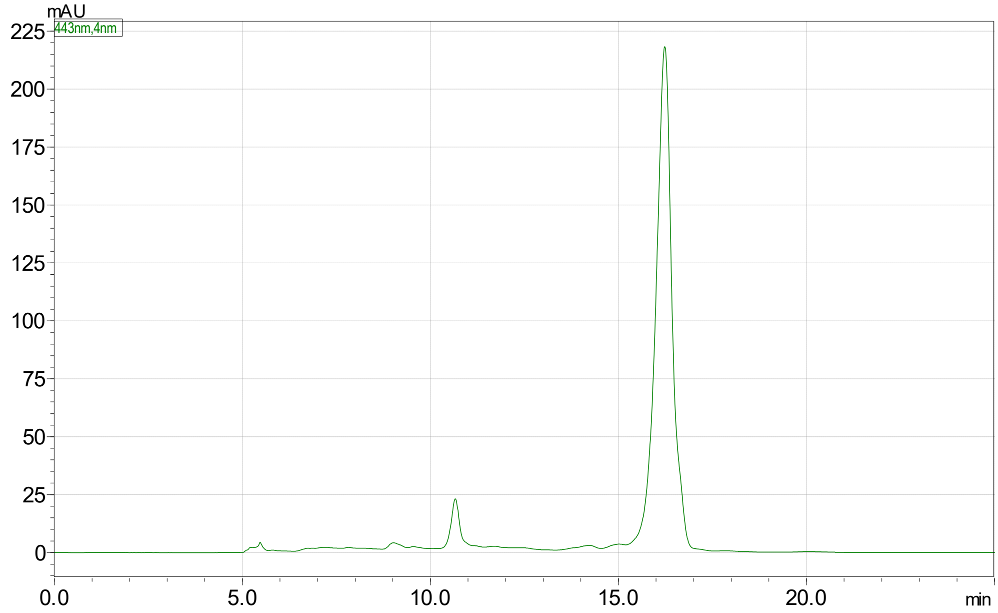

a2

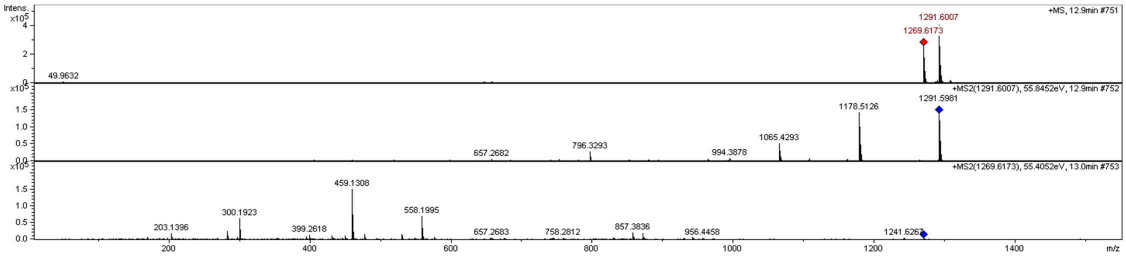

b

Figure S2. HPLC and MS data of pigment a1) All detection wavelengths HPLC (Eluent A: H_2O, Eluent B: Acetonitrile. The isocratic elution (75% B) was in 25 min. Flow rate: 2 ml/min, injection volume: 20 μl); a2) 443nm detection wavelength HPLC; b) MS.

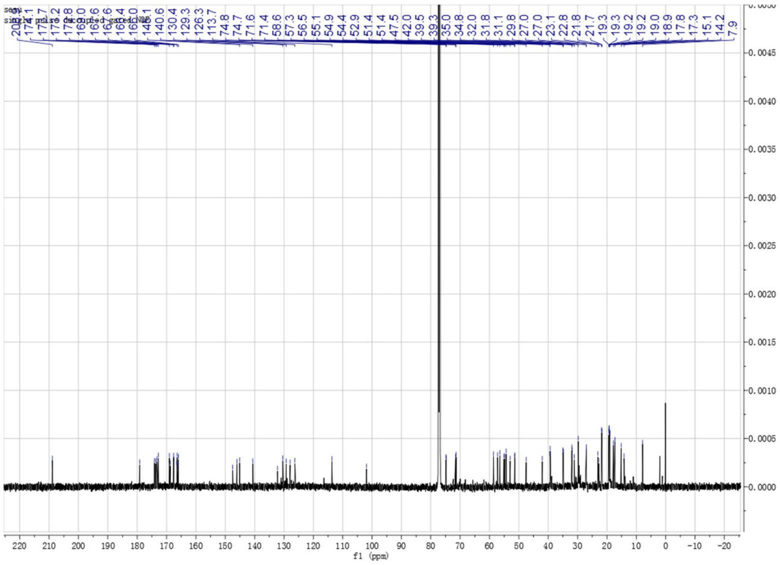

^{13}C-NMR

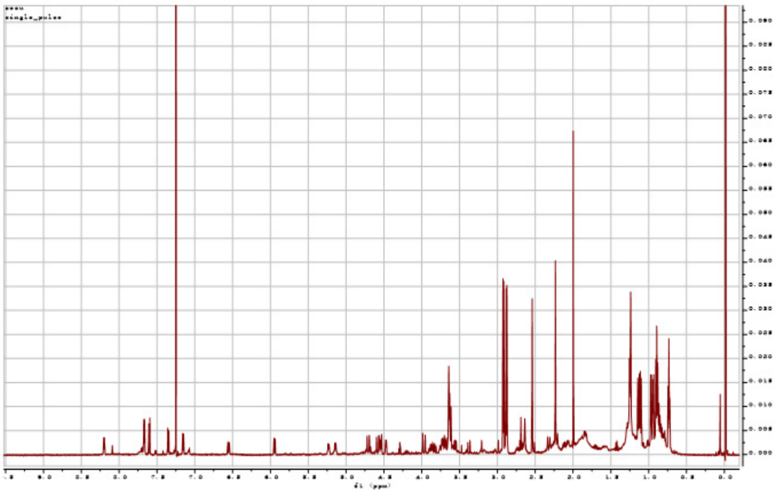

^{1}H-NMR

Figure S3. ^{13}C-NMR and ^{1}H-NMR (at 400 MHz in $CDCl_3$) profiles of the pigment.

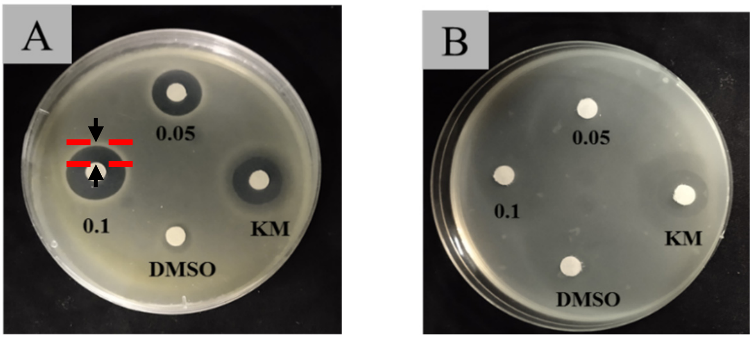

Figure S4. Antimicrobial activity of the pigment to two microorganisms.(Negative control: DMSO Positive control: Kanamycin (KM) 5mg/mL, Ac.X2 pigment concentration: 0.05 mg/mL, 0.1 mg/mL, the zones of inhibition were measured in millimetres.) (A) Staphylococcus aureus, (B) Escherichia coli.

Optimization of pigment production

Pigment yield calculation

The maximum absorption (λ_{max}) of the purified pigment in methanol solution was measured using the spectrophotometer. The maximum ultraviolet absorption under different pigment concentrations was determined to draw the standard curve of pigment concentration concerning ultraviolet absorption. Pigment yield was calculated according to the absorption value of the

pigment at the maximum absorption wavelength after fermentation compared with the standard curve.

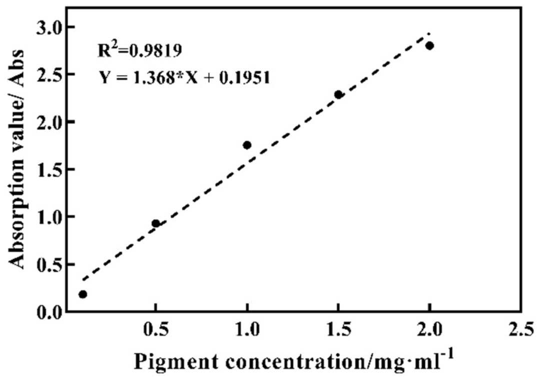

Figure S5. Pigment absorbance standard curve.

The curve of pigment concentration and ultraviolet absorption is shown in Figure S5. Linear regression equation of pigment yield (2):

$$Y = 0.1315X - 0.009167 \quad (2)$$

Where Y is the pigment yield and X is the ultraviolet absorption value. The R squared of the regression equation is 0.9819, and the confidence is over 98%.

Selection of medium, pH, carbon and nitrogen source

Four cultivation conditions were studied for actinomycin X2 production including medium, pH, carbon source and nitrogen source. Firstly, five different liquid media were selected for the fermentation of pigment: ISP1, ISP2, ISP4, ISP5, and PDA 100 mL of each medium was prepared in a 500 mL conical flask. After sterilizing the medium, 1 ml of seed solution was added, and the medium was placed in a 28°C constant temperature shaker and incubated for 7 days at 160 rpm. After the medium with the highest yield of pigment was selected, the initial pH was adjusted to 3.0-9.0. And it follows the carbon source set as malt extract, sucrose, soluble starch, glucose, glycerol, oat powder, and mannitol. The nitrogen source was set as tryptone, casein peptone, yeast extract,

beef extract, ammonium sulfate, malt extract, and soybean powder. Three groups of parallel controls were set for each medium.

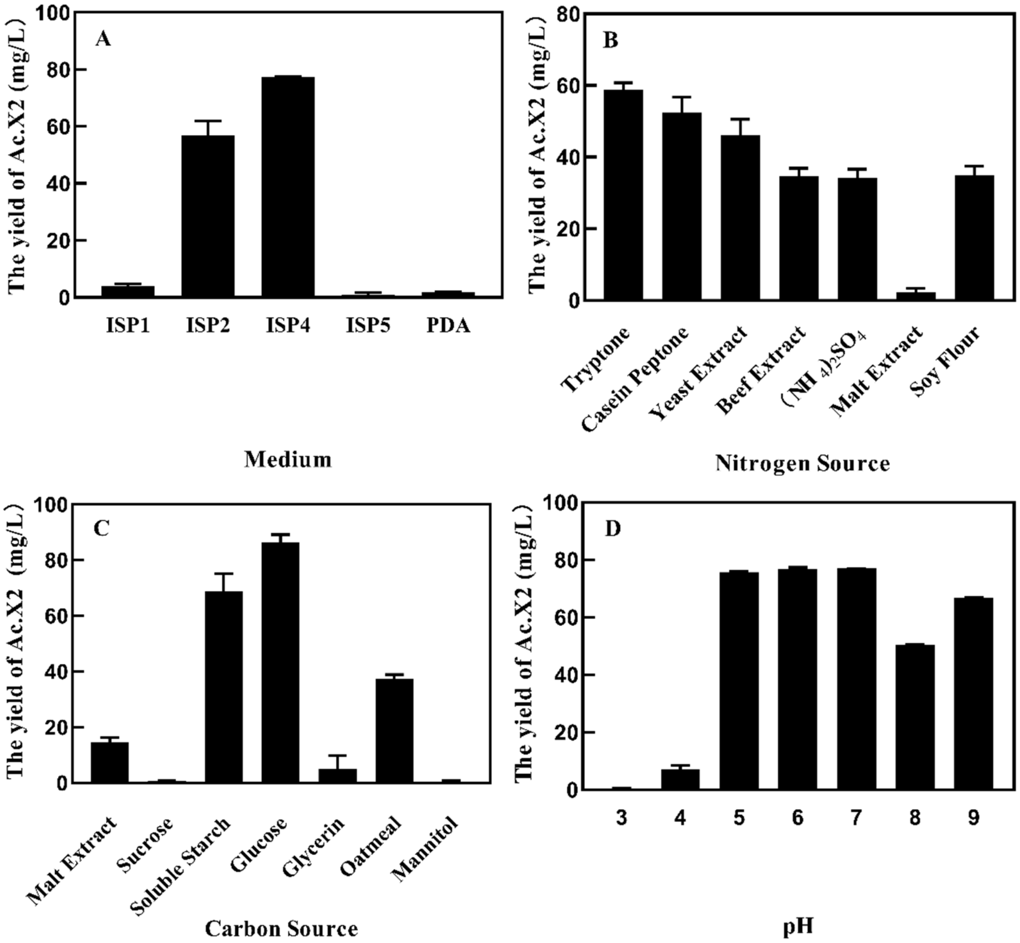

Figure S6. Effects of liquid medium on **(A)** different liquid medium; **(B)** nitrogen source; **(C)** carbon source; **(D)** pH.

Actinomycetes growth and pigment production yields were greatly influenced by cultivation conditions (e.g, cultivation medium, temperature, pH, carbon and nitrogen sources, mineral, aeration, and type of fermentation), and the optimum conditions for pigment production were not homologous for the actinomycetes species and strains. The pigment yield of Ac.X2 in various liquid medium is shown in Fig.S6. The error bar is standard deviations from three independent measurements. The results indicated that the use of ISP4 medium (the carbon and nitrogen sources

were glucose and tryptone, respectively), at the initial pH of 6.0, and cultured at 28°C for 7 days, was the optimum conditions for the pigment production of Ac.X2 strain (98.2 mg/L).

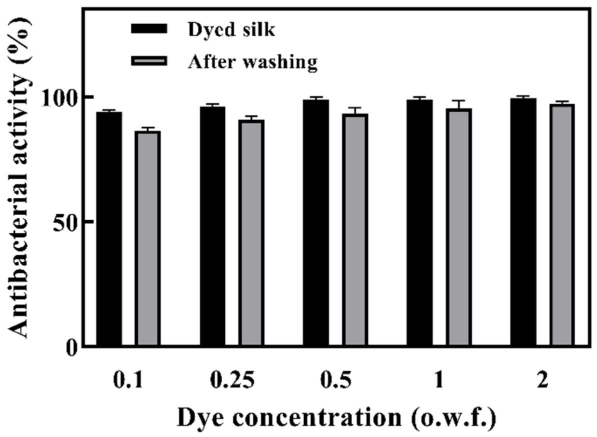

Figure S7. Bacteriostatic rates of different o.w.f silk tested by AATCC 100-2012 method.

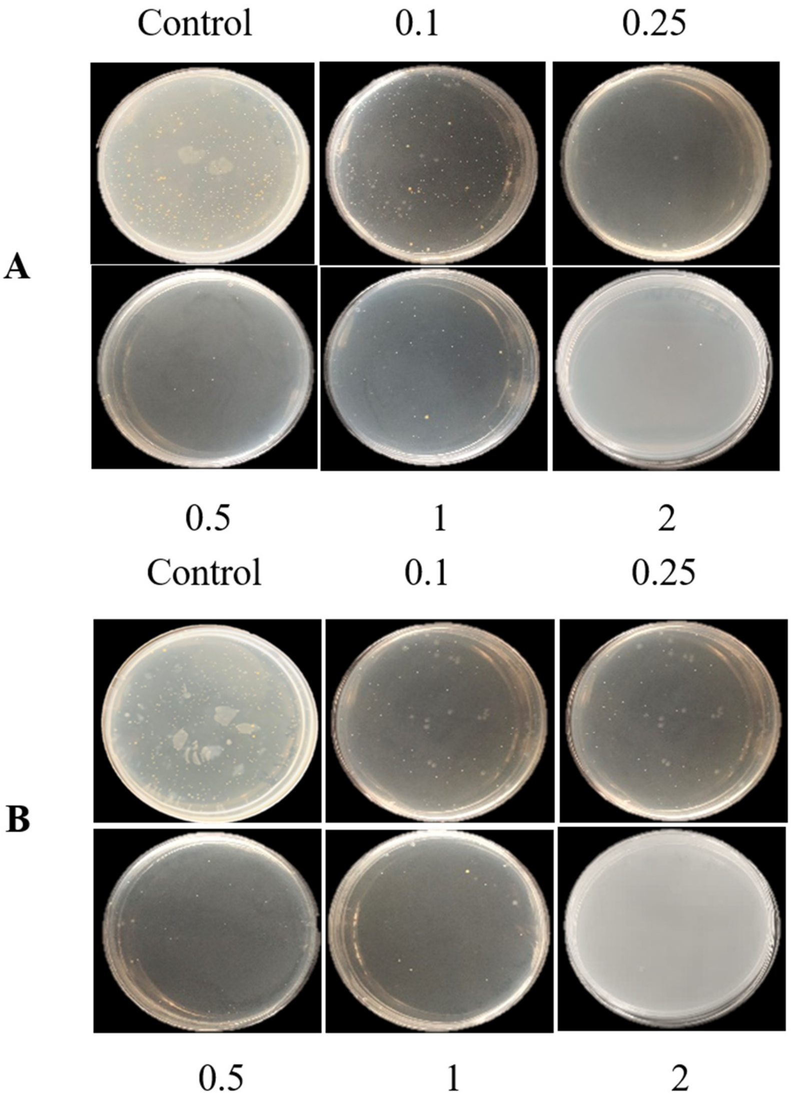

Figure S8. The images of plates of different o.w.f dyed silk against *Staphylococcus aureus* tested by AATCC 100-2012 method(A: Dyed silk; B: After washing five times according to GB/T 12490 A1M method washing five times) (The bacteria solution was diluted by 10-fold to the concentration of 10^{-1}).

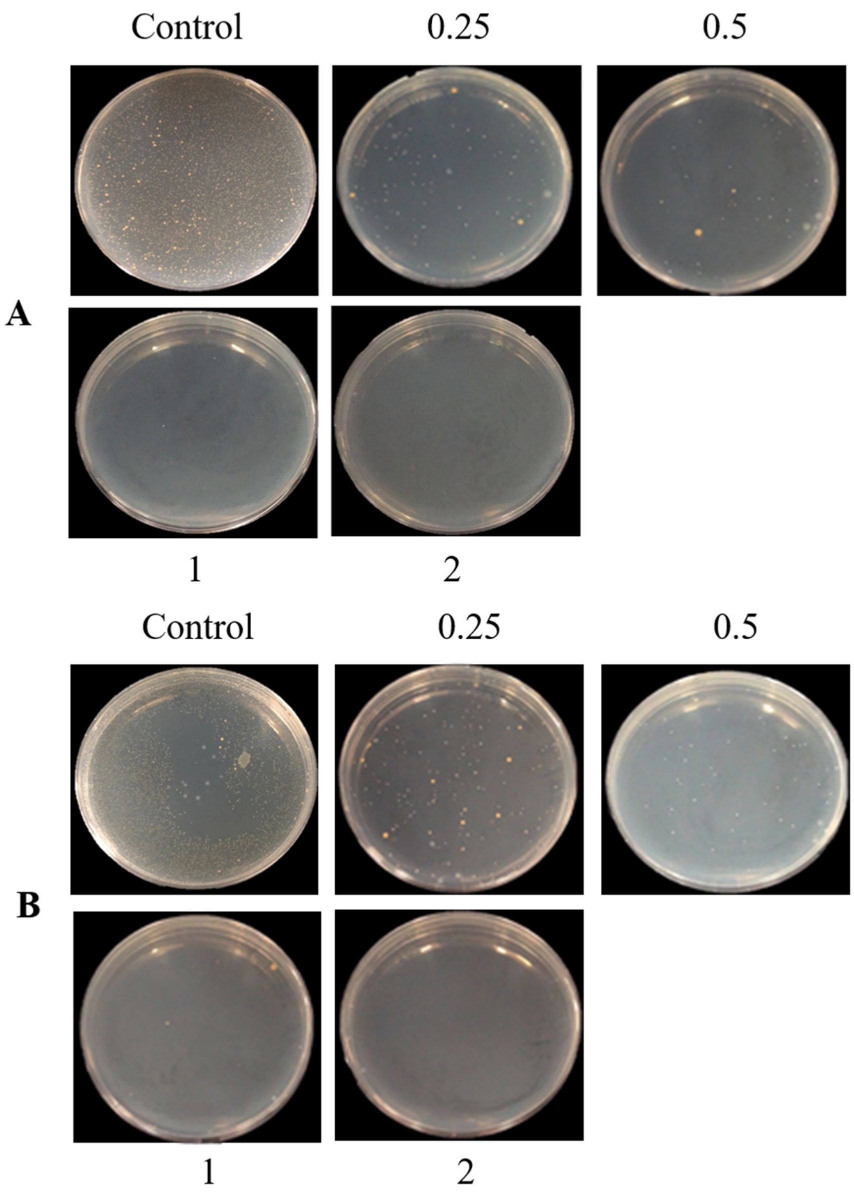

Figure S9. The images of plates of different o.w.f dyed silk against *Staphylococcus aureus* tested by GB/T 20944.3-2008 method(A: Dyed silk; B: After washing five times according to GB/T 12490 A1M method washing five times) (The bacteria solution was diluted by 10-fold to the concentration of 10^{-1}).

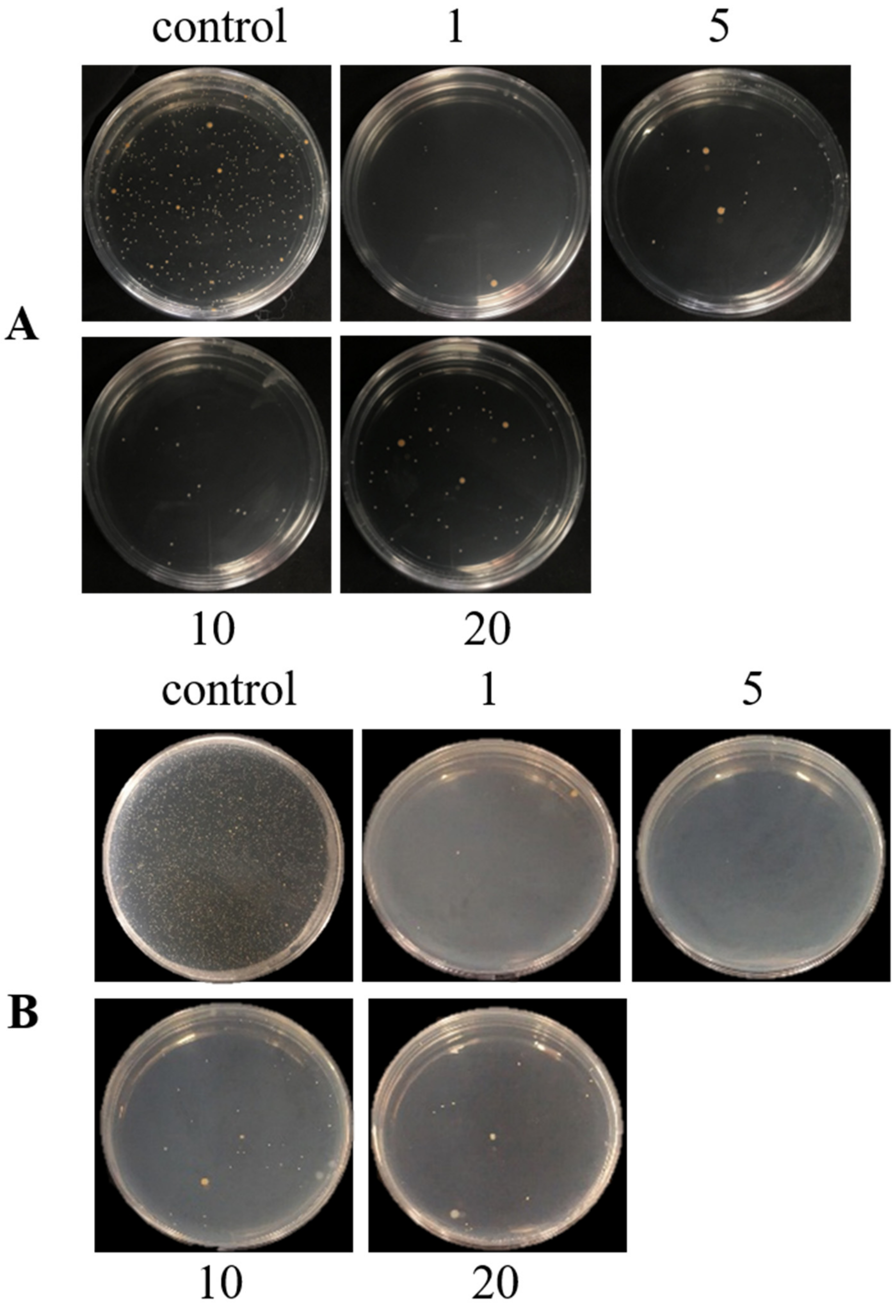

Figure S10. Antibacterial activity of (**A**) dyed o.w. f 0.5% and (**B**) o.w. f 1% silk sample against *Staphylococcus aureus* tested by GB/T 20944.3-2008 method after different washing times.(The bacteria solution was diluted by 10-fold to the concentration of 10^{-1}).

2. Table

Table S1. Antibacterial activity of dyed o.w.f 0.5% and o.w.f 1% silk sample against *Staphylococcus aureus* tested by GB/T 20944.3-2008 method after different washing cycles.

**Washing cycles**	**0.5% (o.w.f) Antibacterial activity**			**0.1% (o.w.f) Antibacterial activity**		
**1**	97.73%	97.73%	97.36%	99.30%	99.30%	99.81%
**5**	96.60%	96.98%	99.05%	98.88%	99.07%	99.30%
**10**	96.22%	94.34%	96.22%	96.65%	97.21%	97.02%
**20**	93.40%	91.69%	90.00%	94.79%	94.97%	94.41%

Unit：CFU/mL
